# Nuclear β-catenin and CD44 upregulation characterize invasive cell populations in non-aggressive MCF-7 breast cancer cells

**DOI:** 10.1186/1471-2407-10-414

**Published:** 2010-08-10

**Authors:** Masahiro Uchino, Hiroko Kojima, Kenta Wada, Mika Imada, Fumitoshi Onoda, Hiroyuki Satofuka, Takahiko Utsugi, Yasufumi Murakami

**Affiliations:** 1 Neo-Technology Development Division, Bio Matrix Research Inc., 105 Higashifukai, Nagareyama, Chiba 270-0101, Japan; 2 Department of Biological Science and Technology, Graduate School of Industrial Science and Technology, Tokyo University of Science, 2641 Yamazaki, Noda, Chiba 278-8510, Japan; 3 Current Address: Technology Development Department, ATTO Corporation, Tokyo 113-0034, Japan; 4 Current Address: Department of Bioproduction, Faculty of Bioindustry, Tokyo University of Agriculture, Hokkaido 099-2493, Japan; 5 Current Address: Pharmaceutical BTO Unit, CAC Corporation, Tokyo 103-0015, Japan

## Abstract

**Background:**

In breast cancer cells, the metastatic cell state is strongly correlated to epithelial-to-mesenchymal transition (EMT) and the CD44^+^/CD24^- ^stem cell phenotype. However, the MCF-7 cell line, which has a luminal epithelial-like phenotype and lacks a CD44^+^/CD24^- ^subpopulation, has rare cell populations with higher Matrigel invasive ability. Thus, what are the potentially important differences between invasive and non-invasive breast cancer cells, and are the differences related to EMT or CD44/CD24 expression?

**Methods:**

Throughout the sequential selection process using Matrigel, we obtained MCF-7-14 cells of opposite migratory and invasive capabilities from MCF-7 cells. Comparative analysis of epithelial and mesenchymal marker expression was performed between parental MCF-7, selected MCF-7-14, and aggressive mesenchymal MDA-MB-231 cells. Furthermore, using microarray expression profiles of these cells, we selected differentially expressed genes for their invasive potential, and performed pathway and network analysis to identify a set of interesting genes, which were evaluated by RT-PCR, flow cytometry or function-blocking antibody treatment.

**Results:**

MCF-7-14 cells had enhanced migratory and invasive ability compared with MCF-7 cells. Although MCF-7-14 cells, similar to MCF-7 cells, expressed E-cadherin but neither vimentin nor fibronectin, β-catenin was expressed not only on the cell membrane but also in the nucleus. Furthermore, using gene expression profiles of MCF-7, MCF-7-14 and MDA-MB-231 cells, we demonstrated that MCF-7-14 cells have alterations in signaling pathways regulating cell migration and identified a set of genes (*PIK3R1*, *SOCS2*, *BMP7*, *CD44 *and *CD24*). Interestingly, MCF-7-14 and its invasive clone CL6 cells displayed increased CD44 expression and downregulated CD24 expression compared with MCF-7 cells. Anti-CD44 antibody treatment significantly decreased cell migration and invasion in both MCF-7-14 and MCF-7-14 CL6 cells as well as MDA-MB-231 cells.

**Conclusions:**

MCF-7-14 cells are a novel model for breast cancer metastasis without requiring constitutive EMT and are categorized as a "metastable phenotype", which can be distinguished from both epithelial and mesenchymal cells. The alterations and characteristics of MCF-7-14 cells, especially nuclear β-catenin and CD44 upregulation, may characterize invasive cell populations in breast cancer.

## Background

Patients with breast cancer are at risk of metastasis throughout their lifetime because of the heterogeneous nature of breast cancer metastasis. Recent studies focusing on the early steps in the metastatic cascade, such as epithelial-to-mesenchymal transition (EMT) and altered cell adhesion and motility, have demonstrated that aggressive cancer progression is correlated with the loss of epithelial characteristics and the gain of a migratory and mesenchymal phenotype [1]. In fact, the highly aggressive breast cancer cell line MDA-MB-231 exhibits mesenchymal-type behavior, whereas non-aggressive breast cancer cell line MCF-7 has a luminal epithelial-like phenotype [2,3].

In addition to the heterogeneous nature of metastasis, a solid tumor including breast cancer is comprised of heterogeneous cells in terms of their invasive and metastatic potential, as suggested by *in vivo *metastasis models [4] and an *in vitro *selection process using Matrigel [5,6]. Tumor heterogeneity has led to the "cancer stem cell hypothesis". Cancer stem cells share common characteristics with normal stem cells: ability to self-renew, differentiate, acquire drug resistance, survive anchorage-independently, and migrate. Furthermore, overlapping sets of molecules and pathways regulate both stem cell migration and cancer metastasis; therefore, cancer stem cells are assumed to contribute to metastasis as well as tumorigenesis [7]. In human breast tumors, the CD44^+^/CD24^-/low ^phenotype has been reported to have stem cell properties [8]. Cell lines with high CD44^+^/CD24^- ^cell numbers were basal/mesenchymal or myoepithelial types and more invasive than other cell lines. In contrast, non-aggressive epithelial MCF-7 cells lack a CD44^+^/CD24^- ^subpopulation. Among CD44^+^/CD24^-^-positive cell lines, MDA-MB-231 has the unique property of expressing a broad range of genes that favor bone and lung metastasis [9]. Although there remains a need to determine whether CD44^+^/CD24^-/low ^cells are true breast cancer stem cells across all the various breast cancer subtypes, there seems to be a connection between EMT and CD44/CD24 expression in the mechanisms of breast cancer invasion and metastasis. Indeed, induction of EMT results in the acquisition of the CD44^high^/CD24^low ^phenotype in immortalized human mammary epithelial cells [10].

In the current study, we demonstrated that non-aggressive epithelial MCF-7 cells had rare cell populations with higher Matrigel invasive ability. Throughout the sequential selection process using Matrigel, we obtained MCF-7-14 cells of opposite migratory and invasive capabilities from MCF-7 cells. Thus, what are the potentially important differences between invasive and non-invasive breast cancer cells, and are the differences related to EMT or CD44/CD24 expression? To answer these questions, comparative analysis of epithelial and mesenchymal marker expression was performed between MCF-7, MCF-7-14, and MDA-MB-231 cells. Furthermore, using microarray expression profiles of these cells, we selected differentially regulated genes for their invasive potential and performed pathway and network analysis to better characterize invasive cell populations in breast cancer.

## Methods

### Cell culture

Human breast cancer cell line MCF-7 was obtained from the Institute of Development, Aging and Cancer, Tohoku University. MDA-MB-231 was purchased from the American Type Culture Collection. Both cell lines were maintained in RPMI 1640 (Sigma-Aldrich, St. Louis, MO) containing 10% fetal bovine serum (FBS) and an antibiotic-antimycotic.

### *In vitro *sequential selection of invasive populations from MCF-7 cells

BD BioCoat Matrigel Invasion Chambers (for a 6-well plate; BD Biosciences, San Jose, CA) were used to select invasive populations from MCF-7 cells. MCF-7 cell suspension (4 × 10^5 ^cells/well) in serum-free RPMI1640 was seeded into the upper chamber. After 60 hours' cultivation, cells that migrated through the membrane (named MCF-7-1) were harvested by trypsinization, proliferated on a dish, and then reseeded in a Matrigel Invasion Chamber. The cycle was repeated 14 times, and finally MCF-7-14 cells were obtained. In each cycle, invading cells on the underside of the chamber were fixed with 10% formalin-neutralized buffer, stained with 0.1% crystal violet, and imaged through a microscope (Axiovert 200M; Carl Zeiss, Jena, Germany) with a CCD camera (AxioCam; Carl Zeiss).

### Cell proliferation assay

MCF-7 and MCF-7-14 cells (5 × 10^3 ^cells/well) were seeded into 96-well plates and cultured. The cells were counted at 24, 48, 72 and 96 h with a Cell Counting Kit-8 (MTT assay; Wako Pure Chemical Industries, Osaka, Japan).

### Western blotting

Cell protein lysates were prepared from confluent cell cultures of MCF-7 and MCF-7-14 cells, subjected to SDS-PAGE, and transferred to PVDF membranes. The membranes were blocked (5% skim milk) and incubated with rat anti-human epidermal growth factor receptor 2, HER-2 (GeneTex, Irvine, CA) and mouse anti-estrogen receptor alpha, ER-α (Stressgen/Assay Designs, Ann Arbor, MI) monoclonal antibodies (mAbs), followed by the corresponding HRP-conjugated secondary antibody (GE Healthcare, Piscataway, NJ). Antibody complexes were detected with the ECL detection system (GE Healthcare).

### *In vitro *wound healing assay

Migratory abilities of MCF-7 and MCF-7-14 cells were measured using the *in vitro *wound healing assay. Cells were plated on 10-cm culture dishes and grown to 100% confluence. Wounds were created by scraping monolayer cells with a sterile pipette tip. At 0, 24, 48, 72 and 96 h after the creation of wounds, three different areas were imaged through an Axiovert 200 M. Wound distances were measured at each time point and expressed as the average percent of wound closure by comparing the zero time.

### Xenografts

Animal experiments were performed in accordance with the Guidelines of the Japanese Government for the Care and Use of Laboratory Animals and approved by the Institutional Animal Care and Use Committee at Tokyo University of Science and the Ethics Committee of Bio Matrix Research Inc. MCF-7 and MCF-7-14 cells were transfected with pEGFP-C1 (encoding the enhanced green fluorescence protein, EGFP; Clontech Laboratories, Mountain View, CA) using Fugene 6 Transfection Reagent (Roche, Basel, Switzerland) in serum-free medium. MCF-7 and MCF-7-14 cells stably transfected with pEGFP-C1 (named MCF-7-EGFP and MCF-7-14-EGFP, respectively) were selected by G418 treatment (500 μg/ml) for 3 weeks. Suspensions of MCF-7-EGFP and MCF-7-14-EGFP cells (1 × 10^6 ^cells/50 μl) were mixed with the same volume of Matrigel, and injected into the fourth mammary fat pad of 10-week-old female BALB/cJ/nu/nu mice.

### *In vivo *and *ex vivo *imaging of EGFP-labeled breast cancer cells

At 4, 8 and 12 weeks after implantation of MCF-7-EGFP and MCF-7-14-EGFP cells, mice were sacrificed and scanned by a luminescence/fluorescence imaging analyzer (LAS3000; Fujifilm, Tokyo, Japan) to detect metastatic lesions. *Ex vivo *fluorescence imaging was also performed on necropsy samples.

### Histology and immunohistochemistry

Xenografts and metastatic lesions were rapidly frozen, and 6 μm thick sections were cut on a cryostat. The sections were analyzed by hematoxylin/eosin (HE) and methyl green pyronin (MGP) staining, or unstained sections were analyzed for EGFP fluorescence. For immunostaining epithelial and mesenchymal markers, mouse anti-cytokeratin (CK) 19 (A53-B/A2; 1:50 dilution; Santa Cruz Biotechnology, Santa Cruz, CA), anti-E-cadherin (67A4; 1:25; Santa Cruz Biotechnology) and anti-vimentin (V9; 1:150; Dako, Glostrup, Denmark) mAbs were used. Briefly, frozen sections were treated with 3% H_2_O_2 _for 10 min and 5% skim milk in Tris-buffered saline with 0.2% Triton-X for 1 h. Primary antibodies were applied for 1 h at room temperature. Subsequently, the sections were incubated with Simple Stain Mouse MAX PO (Nichirei Biosciences, Tokyo, Japan) or Alexa Fluor 488-conjugated goat anti-mouse IgG (1:200; Molecular Probes, Invitrogen, Carlsbad, CA) at room temperature. Diaminobenzidine (DAB) with 0.1% H_2_O_2 _(Dako) was used as the final chromogen and hematoxylin for nuclear counterstaining in immunohistochemistry. In immunofluorescence, cell nuclei were counterstained with 4', 6-diamidino-2-phenylindole (DAPI). Immunostaining of the endothelial marker, CD31, was performed with rat anti-mouse CD31 mAb (MEC 13.3; 1:50; BD Biosciences) and HRP-linked goat anti-rat IgG F(ab')2 fragment (1:200; Amersham ECL, GE Healthcare). Microscopic images were acquired using an Axiovert 200 M equipped with an AxioCam and analyzed by AxioVision software (Carl Zeiss).

### Immunofluorescence staining

MCF-7, MCF-7-14 and MDA-MB-231 cells were plated on coverslips and fixed with 4% paraformaldehyde/phosphate-buffered saline (PBS) for 15 min and permeabilized with 0.1% Triton X-100 in PBS for 2 min, and then incubated in PBS containing 5% skim milk for 1 h at room temperature. Cells were incubated with anti-E-cadherin (1:100), anti-β-catenin (8E7; 1:100; Upstate Biotechnology/Millipore, Billerica, MA), anti-vimentin (1:300) and anti-fibronectin (clone 10; 1:200; BD Biosciences) mAbs for 1 h at room temperature, followed by incubation with Alexa Fluor 488-conjugated goat anti-mouse IgG (1:200-1:1000) and nuclear counterstaining with DAPI.

### RNA isolation

MCF-7, MCF-7-14 and MDA-MB-231 cells were synchronized in S-phase with the thymidine-hydroxyurea block method. Total RNA was isolated from cells arrested in S-phase using an RNeasy Mini kit (Qiagen, Hilden, Germany).

### Microarray analysis

cDNA was synthesized from total RNA isolated from MCF-7, MCF-7-14 and MDA-MB-231 cells using a One-Cycle cDNA Synthesis Kit (Affymetrix, Santa Clara, CA). *In vitro *transcription reactions were performed using a GeneChip IVT Labeling Kit. Fifteen micrograms of the labeled cRNA was hybridized to a Human Genome U133 Plus 2.0 Array (Affymetrix). The array images were scanned and analyzed using Genechip operating software (GCOS; Affymetrix). The full microarray data set is available in the NCBI Gene Expression Omnibus public database under data series accession number GSE18903.

### Microarray data analysis

Using GeneSpring GX 7.3.1 software (Agilent Technologies, Santa Clara, CA), microarray data of each chip were normalized to the 50^th ^percentile of the measurements on that chip. For per-gene normalization, the measurements of each probe were normalized to the median of the measurements of that probe in MCF-7, MCF-7-14 and MDA-MB-231 cell lines. Average linkage hierarchical clustering was carried out on genes filtered on detection flags ("present" in two or more cell lines) and signal intensity (>50 in all cell lines). To identify potentially important differences in biological mechanisms regarding their invasive potential, genes up- or down-regulated >2-fold in both MCF-7-14 and MDA-MB-231 cells compared with MCF-7 cells were selected from the filtered genes. Ingenuity Pathway Analysis software (IPA 5.0; Ingenuity Systems, Redwood City, CA) was also utilized to identify the top significant canonical pathways for these selected genes and to functionally link the most differentially expressed genes.

### Quantitative real-time-PCR

cDNA was synthesized from 2 μg total RNA using SuperScript III reverse transcriptase (Invitrogen). Reverse transcription (RT) was run for 1 h at 50°C and stopped by heating for 5 min at 85°C. PCR was conducted in a 7900HT Fast Real-Time PCR System (Applied Biosystems, Foster City, CA). A 10 μl reaction containing 0.2 μl cDNA, 0.5 μM of each primer and 2.5 μl Power SYBR Green PCR Master Mix (Applied Biosystems) was used to monitor double-strand DNA synthesis. Primer sequences are provided in Additional file 1. Quantitative RT-PCR (qRT-PCR) was carried out following the recommended thermal profile: 95°C for 10 min (pre-incubation) followed by 40 cycles of 95°C for 15 sec (denaturation) and 60°C for 1 min (annealing and elongation). Fluorescence intensity of the amplified products was measured at the end of each PCR cycle. Two runs were performed with each data point run in triplicate. Results were normalized to internal control *GAPDH *mRNA and if necessary, represented relative to mRNA levels of MCF-7 cells.

### Single-cell cloning

MCF-7 and MCF-7-14 cells were plated on 96-well plates at a concentration of a single cell per well, which was confirmed visually. Wells containing either none or more than one cell were excluded from further analysis. Single-cell clones were cultured to allow the growth of individual colonies, which were picked and expanded in culture, and analyzed by Matrigel invasion assay and flow cytometry.

### Flow cytometry

Three human breast cancer cell lines, MCF-7, MCF-7-14 and MDA-MB-231, and single-cell clones derived from the MCF-7 and MCF-7-14 cell lines were used in flow cytometry analysis. Cells were harvested with TrypLE Express (Invitrogen), and then suspended (2 × 10^6 ^cells/100 μl) with Stain Buffer containing 1% FBS (BD Biosciences). Phycoerythrin (PE)-conjugated mAbs against human CD44 (G44-26; BD Biosciences) or CD24 (ML5; BD Biosciences) were added to the cell suspension at the concentrations recommended by the manufacturer and incubated at 4°C in the dark for 60 min. The labeled cells were fixed in 100% methanol on ice for 5 min. Flow cytometry analysis were performed in triplicate using a FACSCalibur system (BD Biosciences).

### Function-blocking antibody treatment

Cells (1 × 10^6 ^cells) were incubated with rat anti-human CD44 mAb (IM7; BD Biosciences) or normal rat IgG at 400 μg/ml for 20 min. After preincubation, a cell proliferation assay, cell migration assay and Matrigel invasion assay were performed. Cell proliferation was assessed by MTT assay. The cell migration assay was performed using an Oris Cell Migration Assay (Platypus Technologies, Madison, WI), which comprises a 96-well plate with silicone stoppers (2 mm diameter) in each well. Following cell seeding and cell attachment (2 × 10^4 ^cells), the stoppers were removed and migration proceeded for 72 h. Cells were then stained with crystal violet and imaged through a microscope. Matrigel Invasion Chambers (for a 24-well plate; BD Biosciences) were used to examine cell invasion. Pretreated cells (5 × 10^4 ^cells) in serum-free RPMI1640 were seeded into the upper chamber and invading cells were fixed and stained after 60 h cultivation.

### Statistical analysis

Data are the means ± standard deviation (SD). Values were compared between MCF-7 and MCF-7-14 cells and between normal IgG and anti-CD44 mAb using Student's *t *test. Comparisons among cell lines were made using analysis of variance (ANOVA) and Tukey-Kramer multiple comparison, where appropriate. *P *< 0.05 was considered significant.

## Results

### Selection and morphological characterization of MCF-7-14 cells

**Figure 1:**
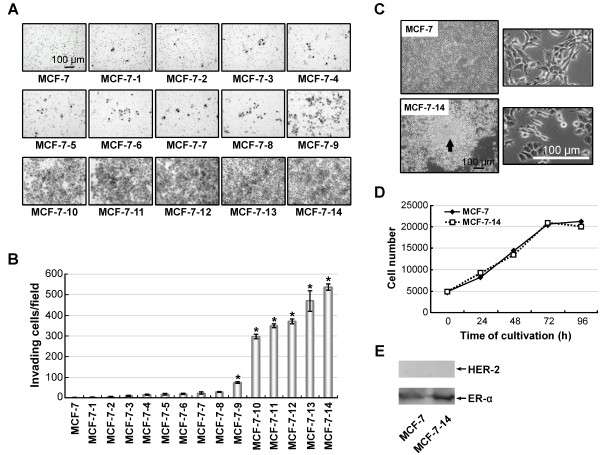
***In vitro *sequential selection of invasive populations from MCF-7 cells using Matrigel Invasion Chambers**. (A and B) In each cycle, cells invading the Matrigel were fixed with 10% formalin-neutralized buffer and stained with crystal violet on the underside of the chamber (A), and then the numbers of invading cells were counted under a microscope (B). Data are the means ± SD (*n *= 3). Statistical analysis was performed by Student's *t *test. *, *P *< 0.01, compared with MCF-7 cells. (C) Images of morphology of MCF-7 and MCF-7-14 cells in culture. MCF-7-14 cells were propagated as non-adherent spheres (arrow). (D) Proliferative rates of MCF-7 and MCF-7-14 cells. (E) Western blotting analysis for the expression of HER-2 and ER-α in MCF-7 and MCF-7-14 cells.

MCF-7 cells were seeded into a Matrigel Invasion Chamber. After 60 hours' cultivation, cells that migrated through the Matrigel membrane (MCF-7-1) were harvested, proliferated on a dish, and then reseeded in a Matrigel Invasion Chamber. Matrigel invasive cells are rare populations within MCF-7 cells. Very few cells migrated through the Matrigel until the 8^th ^cycle of sequential selection (Figure 1A and 1B); however, after the 9^th ^cycle, Matrigel invasive cells markedly increased, and finally, through 14 cycles of sequential Matrigel screening, we obtained MCF-7-14 cells with a significantly (*P *< 0.01) higher proportion of invasive cells than parental MCF-7 cells (Figure 1A and 1B). The selected MCF-7-14 cells in culture were compared with parental MCF-7 cells for morphology and proliferative capacity. MCF-7-14 cells uniquely displayed anchorage-independent growth and formed non-adherent spheres (Figure 1C); however, the proliferative capacity of MCF-7-14 cells was similar to that of parental MCF-7 cells (Figure 1D). In addition, MCF-7-14 cells expressed ER-α but not HER-2, similar to MCF-7 cells (Figure 1E).

### Migratory and metastatic ability of MCF-7-14 cells

**Figure 2:**
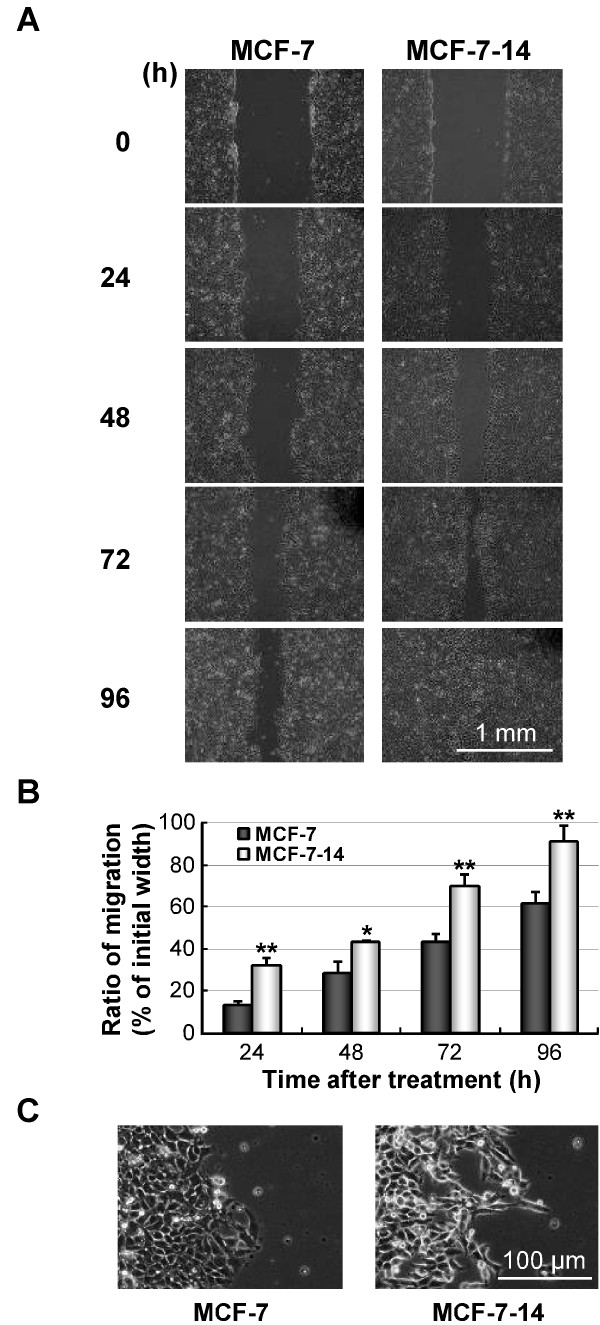
**Migratory abilities of MCF-7 and MCF-7-14 cells in wound healing assay**. (A) Optical microscopic images of *in vitro *wound healing at 0, 24, 48, 72 and 96 h after the creation of wounds. (B) Comparison of images to quantify the migration rate of the cells. Wound distances were measured at each time point and expressed as percent wound closure by comparing the zero time. Data are the means ± SD (*n *= 3). Statistical analysis was performed by Student's *t *test. *, *P *< 0.05; *P *< 0.01, compared with MCF-7. (C) Optical microscopic images of MCF-7 and MCF-7-14 cells at the invading edges of the wound at 48 h after the creation of wounds.

**Figure 3:**
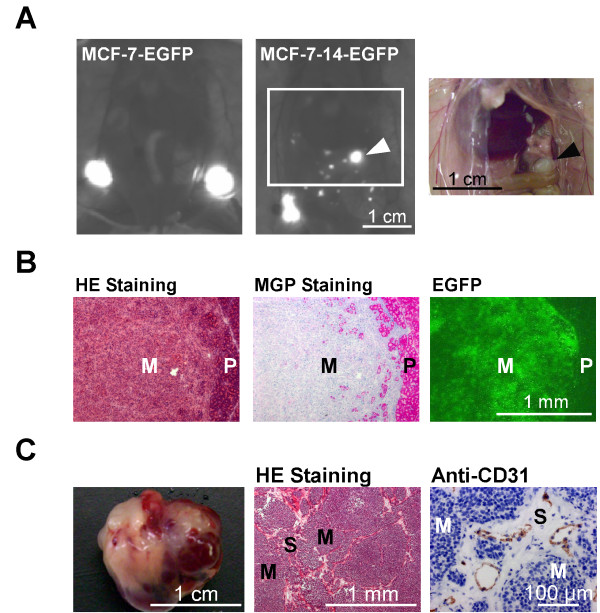
**Metastatic abilities of MCF-7 and MCF-7-14 cells in mouse xenograft model**. (A) *In vivo *scanning of EGFP-expressing breast cancer cells in mice at 4 weeks after implantation into the mammary fat pad. In an MCF-7-14-EGFP-bearing mouse, metastasis to the pancreas (arrowhead) was imaged, corresponding to the photo of the abdomen. (B) EGFP-positive region in panel A was analyzed by HE and MGP staining, or unstained sections were analyzed for EGFP fluorescence. Metastasized MCF-7-14-EGFP cells (M) invaded pancreatic parenchyma (P). (C) Representative pancreas metastasis 12 weeks after implantation of MCF7-14-EGFP cells. Pancreas metastasis showed strong stromal (S) and angiogenic responses (brown) closely intermingled with metastasized tumor cells (M).

The migratory ability of MCF-7-14 cells was compared with that of parental MCF-7 cells using an *in vitro *wound healing assay. MCF-7-14 cells displayed significantly more rapid wound closure at all time points (Figure 2A and 2B), tending to protrude into the wound site, compared with MCF-7 cells (Figure 2C). Using a nude mouse orthotopic xenograft model, we assessed the *in vivo *metastatic ability of MCF-7-14-EGFP cells (Figure 3A). Distant metastases to the pancreas and peritoneum became detectable by fluorescence imaging 4 weeks after implanting MCF-7-14-EGFP cells and appeared in 20% of implanted animals (2/10), whereas no metastatic lesions were detected in MCF-7-EGFP-bearing animals (0/10). Metastasized MCF-7-14-EGFP cells invaded the pancreatic parenchyma, as shown by HE and MGP staining, and expressed EGFP (Figure 3B). In another case, 12 weeks after implantation of MCF-7-14-EGFP cells, pancreas metastasis showed strong stromal and angiogenic responses closely intermingled with metastasized tumor cells, as demonstrated by immunostaining for CD31 (Figure 3C).

### Epithelial and mesenchymal marker expression in MCF-7-14 cells

**Figure 4:**
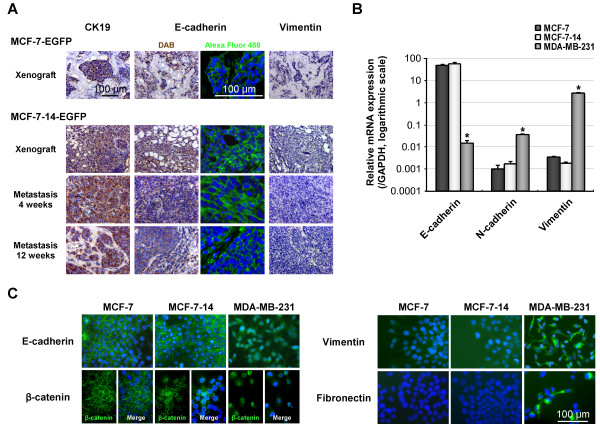
**Comparative analysis of epithelial and mesenchymal marker expression**. (A) Immunostaining for CK19, E-cadherin and vimentin (brown or green) was performed on mammary xenografts and pancreas metastases 4 or 12 weeks after implantation of MCF-7-EGFP and MCF-7-14-EGFP cells. (B) qRT-PCR analysis of mRNA expression of E-cadherin, N-cadherin, and vimentin performed on RNA samples isolated from MCF-7, MCF-7-14 and MDA-MB-231 cells. Results were normalized to internal control *GAPDH *mRNA. Data are the means ± SD (*n *= 3). Statistical analysis was performed by ANOVA and Tukey-Kramer multiple comparison. *, *P *< 0.01, compared with MCF-7 cells. (C) Immunofluorescence analysis of MCF-7, MCF-7-14 and MDA-MB-231 cells using anti-E-cadherin, anti-β-catenin, anti-vimentin and anti-fibronectin mAbs (green). Cells were nuclear counterstained with DAPI (blue).

In immunohistochemistry, mammary xenografts and pancreas metastases generated by MCF-7-14-EGFP cells were positive for a luminal epithelial marker, CK 19, and an epithelial marker, E-cadherin, but negative for a mesenchymal marker, vimentin, similarly to MCF-7-EGFP xenografts (Figure 4A). To molecularly characterize MCF-7, MCF-7-14 and MDA-MB-231 cells in more detail, we analyzed mRNA and protein expressions of epithelial and mesenchymal markers related to EMT. The mRNA expression levels of E-cadherin and mesenchymal markers, N-cadherin and vimentin, were similar between MCF-7 and MCF-7-14 cells, while they confirmed that MDA-MB-231 cells had already undergone "complete EMT", defined by loss of epithelial markers and gain of mesenchymal markers (Figure 4B). Immunofluorescence analysis (Figure 4C) showed the localization of E-cadherin in adherent cell-cell junctions in both MCF-7 and MCF-7-14 cells, whereas E-cadherin was not detectable in MDA-MB-231 cells. In MCF-7 cells, immunofluorescence staining of β-catenin was strongly positive on the cell membrane, whereas in MDA-MB-231 cells, β-catenin was mainly expressed in the nucleus. Interestingly, β-catenin was expressed not only on the cell membrane but also in the nucleus in MCF-7-14 cells. Immunofluorescence staining for vimentin and fibronectin demonstrated cytoplasmic localization in MDA-MB-231 cells, although they were detectable in neither MCF-7 nor MCF-7-14 cells.

### 144 unique genes differentially expressed in both MCF-7-14 and MDA-MB-231 cells

**Figure 5:**
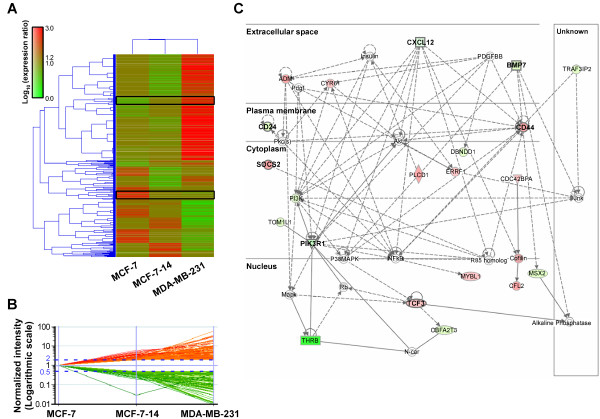
**Microarray gene expression data mining: hierarchical clustering and functional network analysis**. (A) Hierarchical clustering analysis of gene expression profiles in MCF-7, MCF-7-14 and MDA-MB-231 cell lines. Columns represent the 13,092 probe sets detected in all samples. Measurements of each probe were normalized to the median of the probe measurements in MCF-7, MCF-7-14 and MDA-MB-231 cell lines. The intensity of bar colors indicates the degree of gene upregulation (red) or downregulation (green). Although the MCF-7-14 cell gene expression profile was more similar to that of MCF-7 cells than to MDA-MB-231 cells, there were some similarities between MCF-7-14 and MDA-MB-231 cells in gene expression (black flames). (B) To identify potentially important differences in biological mechanisms regarding their invasive potential, we selected 163 probe sets up- (red, see Additional file 2) or downregulated (green, see Additional file 3) >2-fold in both MCF-7-14 and MDA-MB-231 cells compared with MCF-7 cells. Results are represented relative to mRNA levels of MCF-7 cells. (C) The most significant network of 163 probe sets (144 unique genes) constructed using IPA 5.0 (see Additional file 4). Gene network is represented as nodes and lines between two nodes. Node shapes symbolize the functional class of the gene product: square, cytokine; diamond, enzyme; inverted triangle, kinase; rectangle, nuclear receptor; ellipse, transcription regulator; circle, other. The intensity of node colors indicates the degree of upregulation (red) or downregulation (green). Continuous and dashed lines indicate direct and indirect interactions between molecules, respectively. Bold nodes represent multiple-mapped genes (see also Table 1) or selected interesting genes.

To identify genes associated with the invasive phenotype of breast cancer cells, we performed DNA microarray analysis and compared gene expression profiles among MCF-7, MCF-7-14 and MDA-MB-231 cells; 13,092 probe sets were detected in all samples. Hierarchical clustering based on informative probe sets showed that the MCF-7-14 cell gene expression profile was more similar to that of MCF-7 cells than MDA-MB-231 cells; however, there were some similarities between MCF-7-14 and MDA-MB-231 cells in gene expression (Figure 5A). Regarding their invasive potential, we selected 163 probe sets corresponding to 144 unique genes upregulated (76 probe sets, Additional file 2) or downregulated (87 probe sets, Additional file 3) >2-fold in both MCF-7-14 and MDA-MB-231 cells compared with MCF-7 cells from the filtered genes (Figure 5B).

### Top canonical pathways and functional networks for differentially expressed genes

To assess the relevance of the 163 probe sets to previously defined signaling pathways, canonical pathway analysis was performed using IPA 5.0. Table 1 shows the top 16 signaling pathways for the selected genes. Some of these signaling pathways were involved in regulating cell migration and stem cell function. In this analysis, many of the selected genes involved in the top canonical pathways were multiple-mapped genes (9 genes, 74%; Table 1). Considering the general notion that cancer metastasis is a result of multifactorial crosstalk between different molecular pathways, we generated significant functional association networks in invasive cells (see Additional file 4). The network with the highest score (Figure 5C) was constructed relating to DNA replication, recombination and repair, cell cycle, and cancer. Of note, six multiple-mapped genes (*PIK3R1*, *SOCS2*, *BMP7*, *CXCL12*, *CD44 *and *TCF3*; Table 1) were mapped on the most significant network (Figure 5C). These genes were thought to be highly associated with the migratory and invasive phenotype.

**Table 1:** Top signaling pathways for the 163 differentially expressed probe sets in MCF-7-14 and MDA-MB-231 cells.

Pathway	Gene mapped to canonical pathways
JAK/STAT Signaling	***PIK3R1***, ***SOCS2***
Xenobiotic Metabolism Signaling	**ALDH3B2**, **MAOB**, ***PIK3R1***, UGT1A6
Axonal Guidance Signaling	***BMP7***, CFL2, ***CXCL12***, ***PIK3R1***, SEMA6D
TGF-β Signaling	***BMP7***, SMURF2
Acute Phase Response Signaling	***PIK3R1***, ***SOCS2***, ***TCF3***
Leukocyte Extravasation Signaling	***CD44***, ***CXCL12***, ***PIK3R1***
Neuregulin Signaling	ERRFI1, ***PIK3R1***
IGF-1 Signaling	CYR61, ***PIK3R1***
p53 Signaling	***PIK3R1***, **RPRM**
LPS/IL-1 Mediated Inhibition of RXR Function	**ALDH3B2**, **MAOB**, SLC27A2
Serotonin Receptor Signaling	**MAOB**
Notch Signaling	LFNG
Coagulation System	F12
Cell Cycle: G2/M DNA Damage Checkpoint Regulation	**RPRM**
EGF Signaling	***PIK3R1***
Wnt/β-catenin Signaling	***CD44***, ***TCF3***

Pathway analysis was performed on 163 probe sets (144 unique genes) up- or down-regulated >2-fold in both MCF-7-14 and MDA-MB-231 cells compared with MCF-7 cells (see Additional files 2 and 3) using IPA 5.0. Genes in bold are mapped to multiple top canonical pathways, and genes in bold italics are also mapped on the most significant functional network for the 163 probe sets (see also Figure 5C).

### Evaluation of selected interesting genes: CD44 upregulation and CD24 downregulation in MCF-7-14 cells

**Figure 6:**
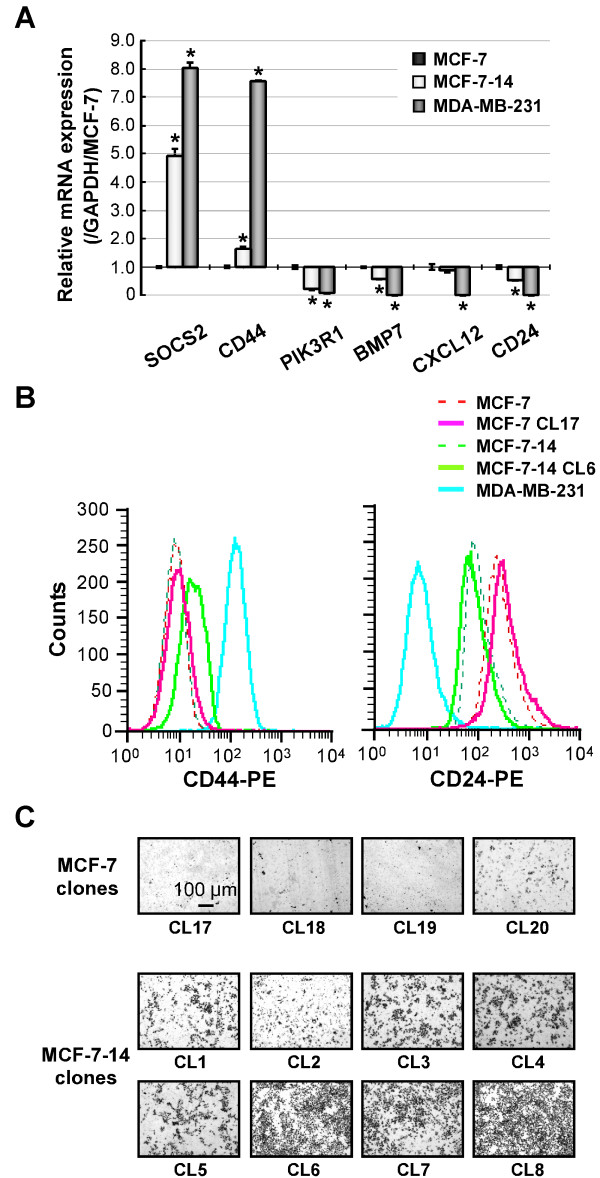
**mRNA and protein expression of selected interesting genes**. (A) qRT-PCR analysis of mRNA expression of *SOCS2*, *CD44*, *PIK3R1*, *BMP7*, *CXCL12 *and *CD24 *was performed on RNA from MCF-7, MCF-7-14 and MDA-MB-231 cells. Results were normalized to internal control *GAPDH *mRNA and represented relative to mRNA levels of MCF-7 cells. Data are the means ± SD (*n *= 3). Statistical analysis was performed by ANOVA and Tukey-Kramer multiple comparison.*, *P *< 0.01, compared with MCF-7 cells. (B) CD44 and CD24 proteins expressed on three human breast cancer cell lines (MCF-7, MCF-7-14 and MDA-MB-231) and single-cell clones derived from MCF-7 and MCF-7-14 cell lines (highly invasive MCF-7-14 CL6 and non-invasive MCF-7 CL17, panel C) were analyzed by flow cytometry. (C) Matrigel invasion assay of single-cell clones derived from MCF-7 (CL17-20) and MCF-7-14 (CL1-8) cell lines. Cells invading Matrigel were stained with crystal violet on the underside of the chamber.

Selected interesting genes (*PIK3R1*, *SOCS2*, *BMP7*, *CXCL12*, *CD44 *and *CD24*) were evaluated by qRT-PCR using total RNA from MCF-7, MCF-7-14 and MDA-MB-231 cells. The mRNA expression levels of *SOCS2 *and *CD44 *were significantly (*P *< 0.01) higher in both MCF-7-14 and MDA-MB-231 cells than in MCF-7 cells. In contrast, *PIK3R1*, *BMP7 *and *CD24 *were significantly (*P *< 0.01) downregulated in MCF-7-14 and MDA-MB-231 cells (Figure 6A); however, MCF-7-14 cells showed a lower degree of upregulation or downregulation than MDA-MB-231 cells. *CXCL12 *mRNA expression was similar between MCF-7 and MCF-7-14 cells, although it was significantly (*P *< 0.01) downregulated in MDA-MB-231 cells. Furthermore, the protein expression levels of CD44 and CD24 were analyzed by flow cytometry. MCF-7 cells expressed low levels of CD44 and high levels of CD24, whereas MDA-MB-231 cells expressed high levels of CD44 and low levels of CD24 (Figure 6B), consistent with the results of qRT-PCR; however, MCF-7-14 cells showed similar levels of CD44 protein expression to MCF-7 cells (Figure 6B, left panel). Thus, single-cell clones were obtained from MCF-7 and MCF-7-14 cells, and analyzed by Matrigel invasion assay and flow cytometry. Single-cell clones derived from MCF-7-14 cells had varying invasive abilities (Figure 6C). The highly invasive cell clone (MCF-7-14 CL6, Figure 6C) displayed a higher expression of CD44 than MCF-7 and its non-invasive clone cells (Figure 6B, left panel). CD24 protein was downregulated in both MCF-7-14 and CL6 cells, compared with MCF-7 and CL17 cells (Figure 6B, right panel). Therefore, these results raise the possibility that alterations in the expression levels of genes selected by pathway and network analysis, including expression patterns of CD44 and CD24, may be accompanied by the acquisition of invasive potential in MCF-7-14 cells.

### Effects of anti-CD44 antibody treatment on cell migration and invasion

**Figure 7:**
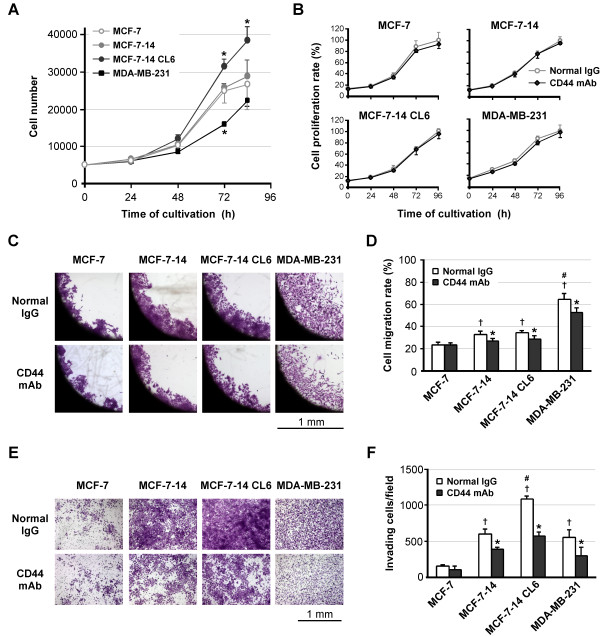
**Effects of anti-CD44 antibody treatment on cell proliferation, migration and invasion**. (A) Proliferation rates of untreated cells in culture. Data are the means ± SD (*n *= 3). Statistical analysis was performed by ANOVA and Tukey-Kramer multiple comparison. *, *P *< 0.01, compared with MCF-7 cells. (B) Proliferation rates of normal rat IgG-treated and anti-CD44 antibody-treated cells. In each cell line, the number of normal IgG-treated cells at 96 h was considered 100%. Data are the means ± SD (*n *= 3). (C) Optical microscopic images of cell migration assay at 72 h after removal of the stopper. Cells were fixed and stained with crystal violet. (D) Cell migration rates were expressed as percent closure of the open area by comparing the zero time. Data are the means ± SD (*n *= 6). Statistical analysis was performed by Student's *t *test or ANOVA/Tukey-Kramer multiple comparison, where appropriate. *, *P *< 0.01, compared with normal IgG-treated cells.^†^, *P *< 0.01, compared with MCF-7.^#^, *P *< 0.01, compared with MCF-7-14 and its clone CL6. (E) Matrigel invasion assay of normal rat IgG-treated and anti-CD44 antibody-treated cells. After 60 h cultivation, cells invading Matrigel were fixed and stained on the underside of the chamber. (F) The numbers of invading cells were counted under a microscope. Data are the means ± SD (*n *= 3). Statistical analysis was performed by Student's *t *test or ANOVA/Tukey-Kramer multiple comparison, where appropriate. *, *P *< 0.01, compared with normal IgG-treated cells.^†^, *P *< 0.01, compared with MCF-7.^#^, *P *< 0.01, compared with MCF-7-14 and MDA-MB-231.

To clarify whether CD44 positively goes hand in hand with increased invasiveness, we examined the effects of anti-CD44 mAb on cell proliferation, migration and invasion. In the cell proliferation assay in the absence of antibodies (Figure 7A), whereas MCF-7-14, similar to MCF-7 cells, reached a plateau after 72 h, MCF-7-14 CL6 cells did not show a plateau phase, but rather continued to grow at a higher growth rate than MCF-7 and MCF-7-14 cells. On the other hand, MDA-MB-231 cells showed slower growth, but reached the same level as MCF-7 cells by the end of the assay. Despite the differences in the growth rate, preincubation with anti-CD44 mAb had no significant effect on cell proliferation in all cell lines tested, compared with treatment with normal IgG (Figure 7B). In the cell migration assay, MCF-7-14 and its clone CL6 cells migrated as a sheet, whereas MDA-MB-231 cells migrated more rapidly and more individually (Figure 7C). In addition to the difference in the method of cell migration, the CD44 expression level differs between these cells; nevertheless, anti-CD44-mAb significantly (*P *< 0.01) decreased cell migration in both MCF-7-14 and MCF-7-14 CL6 cells as well as MDA-MB-231 cells (Figure 7C and 7D). Anti-CD44-mAb also significantly (*P *< 0.01) inhibited cell invasion in these cells, although MCF-7-14 CL6 cells had a significantly higher number of invading cells than MCF-7-14 and MDA-MB-231 cells, differently from the results of the cell migration assay (Figure 7E and 7F).

## Discussion

### MCF-7-14 cells as a novel model for breast cancer metastasis without requiring constitutive EMT

We obtained MCF-7-14 cells that had a significantly higher proportion of invasive cells throughout the *in vitro *sequential selection process, although parental MCF-7 cells had few Matrigel invasive cells. In addition, we demonstrated that EGFP-labeled MCF-7-14 cells metastasized to the pancreas and peritoneum in a xenograft model. In mouse orthotopic xenograft models of breast cancer, the pancreas seems to be a relatively common metastatic site. When injected into the mammary fat pad of nude mice, N-cadherin-expressing cells, but not control MCF-7 cells, metastasized to the liver, pancreas, salivary gland, omentum, lung, lymph nodes, and lumbar spinal muscle [11]. Murine 4T1 mammary cancer cells (expressing the pcDNA-Neo expression vector as a control; 4T1-Neo cells) implanted into the axillary mammary gland of BALB/c mice metastasized to the mesentery adjacent to the pancreas [12]. Despite differences in invasive ability, MCF-7-14 cells, similar to MCF-7 cells, expressed E-cadherin, but neither vimentin nor fibronectin. In the cell migration assay, MCF-7-14 and its clone CL6 cells moved as a sheet, in contrast to the more individual movements of mesenchymal MDA-MB-231 cells. Cell-sheet movement is a typical feature of epithelial cells during embryonic development and wound healing [13]; therefore, MCF-7-14 cells may be a novel model for breast cancer metastasis without requiring constitutive EMT. Complete and constitutive EMT seems not to be required for breast cancer metastasis [1,14,15]. In particular, Lou *et al. *[16] reported that 4T1 cells, which express E-cadherin and ZO-1, are migratory, invasive, and metastasize to multiple sites, and that 4T1-derived (67NR) cells, which form primary tumors but fail to metastasize, express vimentin and N-cadherin, but not E-cadherin. The metastatic ability of breast cancer cells does not seem to strictly correlate with the genotypic and phenotypic properties of EMT per se. In addition, Tarin *et al. *[17] suggested that the fundamental premise that EMT occurs in real cancers is very much in doubt because of the lack of convincing evidence for the conversion of epithelial cells into mesenchymal cell lineages *in vivo*; however, it is possible that MCF-7 and MCF-7-14 cells can make transient EMT *in vitro *in a reversible fashion. For example, estrogen treatment promoted reversible EMT-like changes and collective motility in ER-positive breast cancer cells [18]. The transitory acquisition of mesenchymal characteristics could explain the difficulty in observing EMT in cancer development.

### Nuclear β-catenin expression and "metastable phenoptype" in MCF-7-14 cells

On the other hand, loss of E-cadherin-β-catenin complex is an important step in the progression of many epithelial malignancies [19]. β-catenin has a dual role in the EMT; it enhances cell-cell adhesion when bound to cadherin complexes in adherens junctions and also functions as a transcriptional coactivator upon entry into the nucleus [20]. Nuclear import of β-catenin is another important player in EMT [21,22]. Indeed, in MDA-MB-231 cells, β-catenin was mainly located in the nucleus, whereas in MCF-7 cells, β-catenin was mostly expressed on the cell membrane. Nuclear β-catenin induces a gene expression pattern favoring tumor invasion and proceeds with transition to the mesenchymal phenotype of epithelial tumor cells [23]. In MCF-7-14 cells, β-catenin was expressed not only on the cell membrane but also in the nucleus; therefore, enhanced invasive potential of MCF-7-14 cells may result from "incomplete" EMT, altering some signaling pathways. Several studies [24,25] have identified a hybrid cell showing both epithelial and mesenchymal traits. These cells are referred to as a "metastable phenotype" and distinguished from both epithelial and mesenchymal cells by characteristics such as residual E-cadherin, nuclear β-catenin and sheet movement [13,26]. According to this categorization, MCF-7-14 cells may be just in a "metastable" cell state.

### Alterations in cell migration signaling in MCF-7-14 cells

Here we analyzed which signaling pathways are associated with the enhanced migratory and invasive potential of MCF-7-14 cells. Canonical pathway analysis using IPA 5.0 identified the top 16 signaling pathways for differentially expressed genes in invasive cells. Some of these signaling pathways are involved in regulating cell migration. JAK/STAT (Janus kinase/signal transducer and activator of transcription) signaling is necessary for border cell migration in the *Drosophila *ovary, suggesting its relevance to the progression of cancer [27]. Axon guidance molecules and neuregulin signaling play critical roles in cancer cell invasion as well as in neuronal migration [28,29]. In addition, leukocytes exhibit the ability to sense and move in the direction of a chemoattractant [30]. Acute-phase response signaling modifies inflammatory responses, thereby contributing to leukocyte extravasation [31]. Directed cell movement or chemotaxis is exhibited during wound healing, angiogenesis, embryonic development, immune function, and during cancer cell metastasis [32]. Pathway and network analysis also identified a set of interesting genes, including *PIK3R1*, *SOCS2 *and *BMP7*. Philp *et al. *[33] reported the presence of somatic mutations in *PIK3R1*, the gene for the p85α regulatory subunit of phosphatidylinositol 3-kinase (PI3K), in primary human colon and ovarian tumors and cancer cell lines, resulting in the constitutive activation of PI3K. In addition, MCF-7-14 and MDA-MB-231 cells displayed the upregulated expression of *SOCS2 *and downregulated expression of *BMP7 *compared with MCF-7 cells. JAK/STAT signaling is negatively regulated by overexpression of SOCS proteins, and SOCS2 interferes with the negative regulatory effects of SOCS1 and SOCS3 [27,34]. SOCS2 was generally upregulated in primary breast tumors that developed bone metastasis [35]. On the other hand, transforming growth factor beta (TGF-β) is a pro-oncogene in the later stages of tumorigenesis and appears to contribute to metastasis [36,37]. BMP7 is an antagonist of the TGF-β pathway and can inhibit osteolytic metastasis attributable to prostate cancer [38]. Decreased BMP7 expression during carcinogenesis in the human breast contributes to the acquisition of a bone metastatic phenotype [39]. PI3K, JAK/STAT and TGF-β signaling play key roles in cell migration, and PIK3R1, SOCS2 and BMP7 are regulatory molecules of these signaling pathways, thereby possibly contributing to metastasis development. Consequently, alterations in these signaling pathways may explain the differences in migratory and invasive potential between MCF-7 and MCF-7-14 cells, demonstrating the validity and usefulness of this comparative analysis.

### Possible relevance of MCF-7-14 cells to cancer stem cells

Canonical pathway analysis identified xenobiotic metabolism signaling as one of the top signaling pathways in invasive cells. This signaling pathway is possibly responsible for maintenance of the stem cell phenotype and multidrug resistance in breast cancer cells [40]. In addition, some of the top signaling pathways (JAK/STAT, TGF-β, epidermal growth factor and Wnt/β-catenin signaling) overlapped the signaling pathways important for cancer stem-like cells, identified between the MCF-7 side population (SP) and non-SP cells [41]. These signaling pathways are involved in both tumorigenesis in cancer and self-renewal in embryonic stem cells [42]. Although there are no direct evidences yet, MCF-7-14 cells, which have been derived from rare cell populations with higher invasive ability within MCF-7 cells, may be possibly relevant to cancer stem-like cells. MCF-7-14 cells have similar morphological characteristics to those of stem/progenitor or chemotherapy-resistant cells isolated from MCF-7 cells. Cells grown as non-adherent spherical clusters, which were isolated from breast cancer lesions or MCF-7, displayed stem/progenitor cell properties [43]. MCF-7 cells that survived chemotherapeutic treatment formed looser colonies in the monolayer, with cells at the borders tending to have more protruding filopodia [44]. Furthermore, invasive populations seem to generate both invasive and non-invasive populations resembling the cases seen in SP [45] and tumorigenic CD44^+^CD24^-/low ^cells [8] of breast tumor cells, because the numbers of cells invading the Matrigel were very low until the 8^th ^cycle of *in vitro *sequential selection. Interestingly, MCF-7-14 cells displayed increased expression of CD44 mRNA and downregulated expression of CD24 mRNA and protein. Although MCF-7-14 cells showed cell population heterogeneity in invasiveness and CD44 protein expression, MCF-7-14-derived clone MCF-7-14 CL6, which had a higher number of invading cells, displayed upregulation of CD44 and rapid and extended proliferation. However, there are no overlapping genes between our results and the gene signature identified by gene expression profiling of CD44^+^CD24^- ^breast cancer stem-like cells [46]; therefore, our findings should be validated in further studies, for example, analysis of stem cell marker expression.

### Nuclear β-catenin and CD44 upregulation characterize invasive cell populations in breast cancer

Mani, Weinberg and colleagues [10] found that cells that had undergone EMT exhibited CD44^high^/CD24^low ^expression, suggesting that the EMT might confer malignant properties on breast tumor cells. Although the EMT seems not to be an "all or nothing" event, upregulation of CD44 and downregulation of CD24 seem to participate in the acquisition of invasive and metastatic potential [47-49]. In this study, despite the difference in CD44 expression, treatment with CD44 mAb (clone IM7), which induces CD44 shedding from the cell surface [50], significantly decreased cell migration and invasion in MCF-7-14 and its clone CL6 cells as well as MDA-MB-231 cells. Although CD44 expression seems to be correlated with mesenchymal marker expression as well as with invasiveness, CD44 may be one of the most important adhesion molecules facilitating cell invasion and metastasis [51]. Furthermore, CD44 expression is, directly or indirectly, regulated by the β-catenin/Tcf-4 signaling pathway, especially in the colorectal cancer precursor lesions, suggesting a role for CD44 in intestinal tumorigenesis [52,53]. Brabletz *et al. *[21] reported that nuclear β-catenin accumulates in dedifferentiated colorectal carcinoma cells at the tumor-host interface, whereas a gradual loss of nuclear β-catenin is seen towards central, well-differentiated areas of the tumor. This heterogeneous pattern of the primary tumor is recapitulated in corresponding liver metastases. Nuclear accumulation of β-catenin in colorectal cancer cells at the invasive front and in the vessels has been suggested to be a powerful predictor of liver metastasis [54]. In breast cancer, Wnt/β-catenin activation is an important feature of basal-like breast cancers and is predictive of worse overall survival [55]. Consequently, nuclear β-catenin and upregulation of CD44 may be potential diagnostic and therapeutic targets for breast cancer metastasis.

## Conclusions

Although the MCF-7 cell line has a luminal epithelial-like phenotype and lacks a CD44^+^/CD24^- ^subpopulation, we obtained MCF-7-14 cells of opposite migratory and invasive capabilities from MCF-7 cells and developed a novel model for breast cancer metastasis without requiring constitutive EMT. This study better characterizes invasive breast cancer cells and provides insight into understanding the biology of breast cancer metastasis. In clinical diagnosis, ER-positive, HER2-negative breast cancer, which is defined as "luminal A" by the specific expression of an intrinsic set of genes [56], is the most common type of breast cancer and tends to have a better prognosis than the other three types (luminal B, HER-2 overexpressing, basal-like). However, the clinical behavior of luminal-type breast cancer can be markedly heterogeneous despite similar levels of ER expression [57]; therefore, a set of genes differentially regulated in MCF-7-14 cells (*PIK3R1*, *SOCS2*, *BMP7*, *CD44 *and *CD24*) may be useful markers to identify among good prognosis tumors those that will relapse and metastasize. MCF-7-14 cells, in particular, showed nuclear β-catenin expression and a similar phenotype to "metastable" cells, which are distinguished from both epithelial and mesenchymal cells [13]. In addition, MCF-7-14 and its invasive clone CL6 cells displayed increased expression of CD44 and downregulated expression of CD24 compared with MCF-7 cells. The alterations and characteristics of MCF-7-14 cells may characterize invasive cell populations in breast cancer.

## Abbreviations

ANOVA: analysis of variance; CK: cytokeratin; DAB: diaminobenzidine; DAPI: 4', 6-diamidino-2-phenylindole; EGFP: enhanced green fluorescence protein; EMT: epithelial-to-mesenchymal transition; ER-α: estrogen receptor alpha; FBS: fetal bovine serum; HE: hematoxylin/eosin; HER-2: human epidermal growth factor receptor 2; JAK/STAT: Janus kinase/signal transducer and activator of transcription; mAb: monoclonal antibody; MGP: methyl green pyronin; PBS: phosphate-buffered saline; PE: phycoerythrin; PI3K: phosphatidylinositol 3-kinase; qRT-PCR: quantitative reverse transcription polymerase chain reaction; SD: standard deviation; SP: side population; TGF-β: transforming growth factor beta.

## Competing interests

MU, HS and TU are employees of Bio Matrix Research Inc. HK and KW are former employees of the company. YM is a co-founder and the COO of the company. MI, HK, MU, TU and YM are co-inventors of a pending patent entitled "MCF7-derived cell" (WO/2008/093886). This is a joint application by Bio Matrix Research Inc. and Tokyo University of Science. MU, HK, TU and YM are also co-inventors of a pending patent entitled "Methods and kits for evaluating malignancy of breast cancer" (PCT/JP2009/070078), which is filed by Bio Matrix Research Inc. FO declares no conflict of interest.

## Authors' contributions

MU, HK and KW participated in the design and coordination of the study, carried out the analyses and wrote the manuscript. MI performed sequential selection of invasive populations from MCF-7 cells and obtained MCF-7-14 cells. HS performed function-blocking experiments and helped draft the manuscript. FO, TU and YM were responsible for the study conception, data interpretation and manuscript preparation. All authors read and approved the final manuscript.

## Pre-publication history

The pre-publication history for this paper can be accessed here:

http://www.biomedcentral.com/1471-2407/10/414/prepub

## Supplementary Material

Additional file 1 **Primer sequences used for qRT-PCR**. Click here for file

Additional file 2 **List of the 76 probe sets upregulated >2-fold in both MCF-7-14 and MDA-MB-231 cells compared with MCF-7 cells**. Click here for file

Additional file 3 **List of the 87 probe sets downregulated >2-fold in both MCF-7-14 and MDA-MB-231 cells compared with MCF-7 cells**. Click here for file

Additional file 4 **Top significant networks for the 163 differentially expressed probe sets (144 unique genes) in both MCF-7-14 and MDA-MB-231 cells compared with MCF-7 cells**. Click here for file
